# Exploratory evaluation of equine placental extract supplementation on ageing indicators in geriatric dogs: a single-arm pre–post study

**DOI:** 10.1186/s12917-026-05475-y

**Published:** 2026-04-15

**Authors:** Naoya Morita, Eiichi Hirano

**Affiliations:** 1 Marketing Department, Japan Bio Products Co, Ltd, Tokyo, Japan; 2 Medical Affairs Department, Japan Bio Products Co, Ltd, Tokyo, Japan

**Keywords:** Placental extract, Geriatric dogs, Dietary supplement, Vitality, Appetite, Pre–post study, Exploratory study

## Abstract

**Background:**

Recent advances in preventive veterinary healthcare, nutritional management, and owner engagement have extended the longevity of domestic dogs. However, this increased lifespan has led to a higher prevalence of age-associated physiological and functional decline. Equine placental extract contains a variety of bioactive constituents, with reported antioxidative and anti-inflammatory properties. Although the use of equine placental extract in veterinary practice is expanding, empirical evidence supporting its effects on ageing-related clinical outcomes in dogs remains limited. In this study, we aimed to evaluate the effects of 28 days of oral equine placental extract supplementation on ageing status, vitality, appetite, and safety in geriatric dogs using a single-group pre–post design. Accordingly, 20 client-owned geriatric dogs aged > 11 years received daily oral equine placental extract supplementation; and longitudinal changes in clinical ageing scores, owner-assessed vitality and appetite, haematological and biochemical parameters, and safety outcomes were evaluated.

**Results:**

Daily equine placental extract supplementation significantly reduced ageing-level assessment scores, with the median (interquartile range) values decreasing from 24.0 (15.0–29.0) at Day 0 to 21.5 (12.0–23.0) at Day 14 and 18.0 (10.5–21.5) at Day 28. Vitality and appetite, assessed using a 10-point visual analogue scale, increased from 5.0 ± 1.2 to 6.7 ± 1.5 and 7.5 ± 2.0, and from 5.3 ± 1.5 to 7.1 ± 1.3 and 8.4 ± 1.7, respectively, across the same timepoints. No significant changes were observed in body weight, rectal temperature, or haematological and biochemical parameters. Two adverse events occurred—one episode of haematuria occurring prior to the intervention and one fatality suspected to be attributable to rodenticide ingestion. Neither was considered related to the intervention. Exploratory physical activity monitoring was conducted; however, no statistical analysis was performed due to the small sample size.

**Conclusions:**

Oral equine placental extract supplementation for 28 days was associated with improvements in ageing-related clinical scores, vitality, and appetite in geriatric dogs. These findings should be interpreted as preliminary and hypothesis-generating, and do not establish efficacy. Equine placental extract may have potential as a supportive intervention for age-associated decline; however, further controlled studies are required. Adequately powered, controlled trials are required to confirm its efficacy and clarify underlying mechanisms.

**Supplementary Information:**

The online version contains supplementary material available at 10.1186/s12917-026-05475-y.

## Background

The lifespans of domestic dogs have increased by approximately 3–4 years in recent decades, driven by improvements in preventive healthcare; including vaccinations, deworming, and dental care; and greater availability of early diagnosis and treatment [1, 2]. This trend is associated with the growing recognition of pets as family members, characterised by high-quality nutrition, indoor living, regular veterinary visits, and increased owner investment in pet care [1, 2]. Consequently, domestic dogs, akin to humans, increasingly experience a range of age-related diseases [3, 4]. Moreover, because they share household environments with their owners—including similar exposure to environmental pollutants, stressors, activity patterns, and dietary habits—dogs often develop clinical signs that closely parallel those observed in humans [5, 6].

From this perspective, the concept of ‘Canine Geriatric Syndrome (CGS)’ has been proposed as a comprehensive model to describe canine ageing [4]. This concept adapts the framework of the human Geriatric Syndrome to dogs, providing a holistic approach for understanding and managing age-related functional decline. Early detection, quantification, and intervention—including systematic screening, functional assessment, and multifaceted care—are central to this model, as is the establishment of priority research and clinical objectives, such as identification of biomarkers, standardisation of functional assessment tools, and controlled intervention trials [4]. Additionally, the CGS guidelines outline specific actions to support healthy ageing, including regular nutritional assessment, rehabilitation, behavioural and cognitive care, and effective pain management [7–10].

In the context of nutritional interventions, omega-3 fatty acids have demonstrated efficacy in supporting cognitive function in dogs with Canine Cognitive Dysfunction and in alleviating osteoarthritis in geriatric dogs [11, 12]. However, ageing is driven by multiple interconnected processes—including cognitive decline, joint disease, loss of muscle mass, impaired immune function, increased oxidative stress, and alterations in renal and hepatic physiology—making it challenging for any single supplement to provide comprehensive prevention. Accordingly, a multimodal supplementation strategy is often preferable. Although options such as omega-3 fatty acids can address specific age-related changes, effective interventions capable of broadly mitigating the overall ageing process in older dogs remain limited.

Placental extract is a bioactive substance derived from placental tissue through enzyme-based extraction and is known to exert anti-inflammatory and antioxidant effects in mammals, including humans, with increasing interest in its veterinary applications [13–15]. These effects are attributed to its diverse bioactive constituents, including peptides, amino acids, and nucleotides, which have been shown to promote antioxidative, anti-inflammatory, and tissue-regenerative responses in human, livestock, and rodent models [14, 16]. Notably, placental extracts enhance the activity of antioxidant enzymes such as superoxide dismutase, catalase, and glutathione peroxidase, while reducing lipid peroxidation markers, including malondialdehyde [14].

Among placental-derived products, equine placental extract (eqPE) has been investigated in several canine clinical studies. For example, eqPE administration reduced excessive nocturnal barking in three geriatric dogs diagnosed with cognitive dysfunction syndrome [17], improved skin and coat condition in dogs with dermatitis [18], and improved liver function in dogs diagnosed with protein-losing enteropathy (PLE) [19]. Collectively, these observations suggest that eqPE may influence age-related physiological changes; however, evidence supporting anti-ageing effects remains limited and inconclusive.

However, the existing literature on the effects of eqPE supplementation in elderly dogs is limited. Therefore, in this study, we aimed to elucidate its effects on ageing-related indicators and broader systemic conditions in geriatric dogs. We evaluated changes in ageing-level assessment scores—a key indicator of age-related decline—following 28 consecutive days of oral eqPE administration. Furthermore, overall condition—specifically vitality and appetite—was assessed using a visual analogue scale (VAS). Haematological parameters and liver- and kidney-function markers were evaluated as part of the safety assessment.

## Results

### Effect of eqPE supplementation on ageing-level assessment scores

**Fig. 1:**
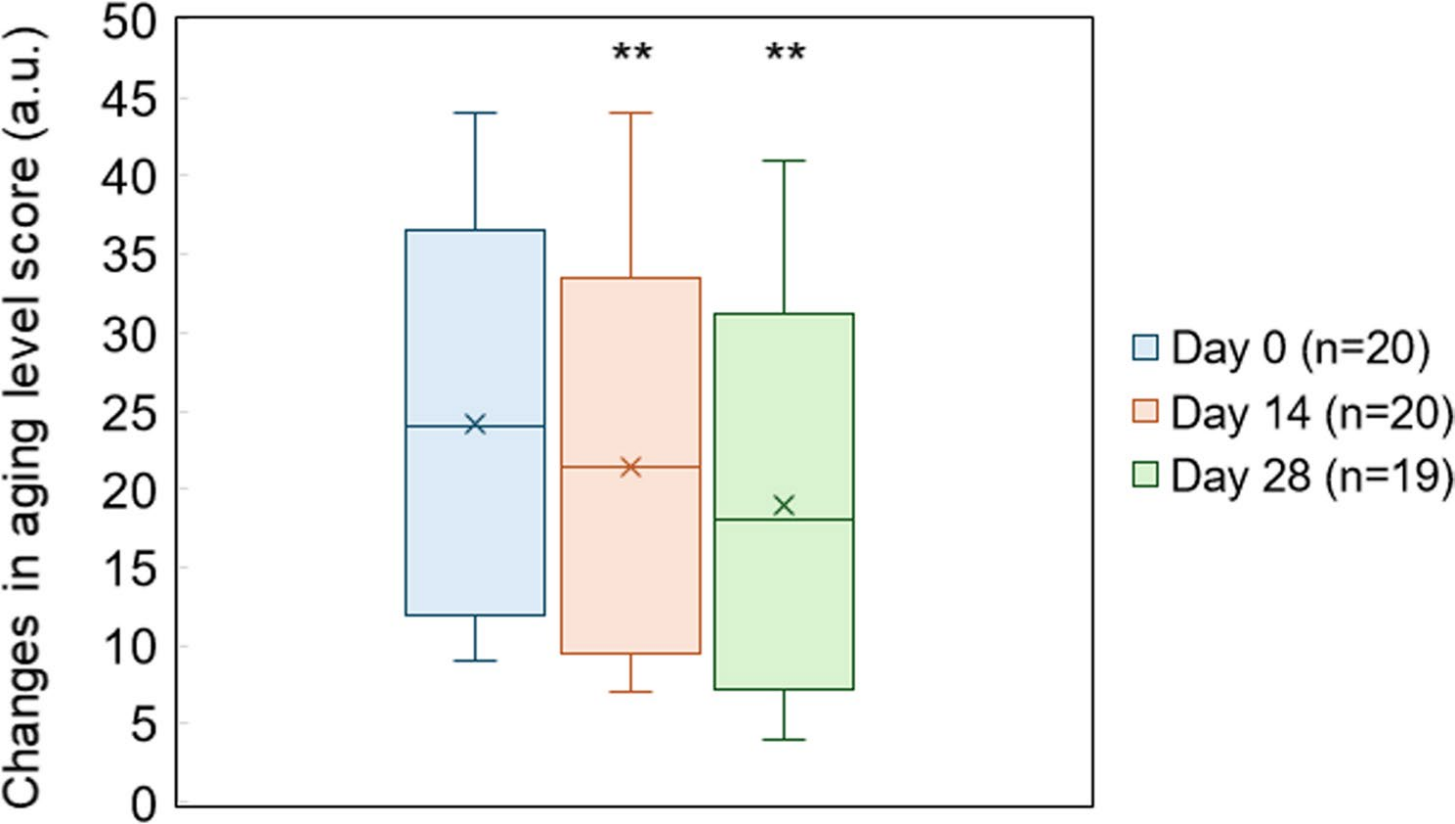
Changes in ageing-level assessment scores across the study period in dogs receiving equine placental extract supplementation. Box plots of total ageing-level assessment scores on Days 0, 14, and 28 of continuous equine placental extract administration. Each box represents the interquartile range (IQR), with the median indicated by the horizontal line. Whiskers extend to 1.5 × IQR, and outliers are shown as individual points. The scores showed a progressive decrease over time, consistent with the statistical results presented in Table 1. ** *p* < 0.01 vs. Day 0

Changes in the comprehensive ageing-level assessment scores are summarized in Table 1. The median (interquartile range, IQR) scores at Day 14 and Day 28 were 21.5 (12.0–23.0) and 18.0 (10.5–21.5), respectively, representing a significant reduction compared to those at Day 0 [24.0 (15.0–29.0)] (Day 14: *p* = 0.013; Day 28: *p* = 0.002) (Table 1; Fig. 1).

**Table 1:** Changin aging level score of the dogs before and after supplementation

	Min	Q1	Med	Q3	Max	*p*-value (vs. Day 0)
Day 0	9.0	15.0	24.0	29.0	44.0	–
Day 14	7.0	12.0	21.5	23.0	44.0	0.013
Day 28	4.0	10.5	18.0	21.5	41.0	0.002

*Min* Minimum, *Q1* First Quartile, *Med* Median, *Q3* Third Quartile, *Max* Maximum, Sample sizes: Day 0 (*n* = 20), Day 14 (*n* = 20), Day 28 (*n* = 19)

### Effect of eqPE supplementation on vitality, appetite, body weight, and rectal temperature

Vitality scores at Day 14 (6.7 ± 1.5; 95% confidence interval (CI): 1.2–2.3), and Day 28 (7.5 ± 2.0; 95% CI: 1.7–3.2) were significantly higher than those at Day 0 (5.0 ± 1.2) (Day 14: *p* < 0.001; Day 28: *p* < 0.001) (Table 2). Appetite scores at Day 14 (7.1 ± 1.3; 95% CI: 1.2–2.5) and Day 28 (8.4 ± 1.7; 95% CI: 2.1–3.9) were also significantly higher than those at Day 0 (5.3 ± 1.5) (Day 14: *p* < 0.001; Day 28: *p* < 0.001) (Table 2). The mean body weight at Day 14 (8.7 ± 3.9 kg; 95% CI: 0.0–0.2) and Day 28 (8.4 ± 4.0 kg; 95% CI: −0.1–0.2) did not differ significantly from that at Day 0 (8.6 ± 3.9 kg) (Day 14: *p* = 0.217; Day 28: *p* = 0.695) (Table 2). Similarly, rectal temperatures at Day 14 (38.0 ± 0.6 °C; 95% CI: −0.1–0.1) and Day 28 (38.1 ± 0.5 °C; 95% CI: −0.1–0.4) did not differ significantly from those at Day 0 (38.0 ± 0.7 °C) (Day 14: *p* = 0.812; Day 28: *p* = 0.163) (Table 2).

**Table 2:** Changin vitality, appetite, body weight and rectal temperature of the dogs before and after supplementation

		Mean ± SD	MD	SE	95% CI	*p*-value (vs. Day 0)
Vitality	Day 0	5.0 ± 1.2	–	–	–	–
	Day 14	6.7 ± 1.5	1.7	0.3	1.2–2.3	< 0.001
	Day 28	7.5 ± 2.0	2.5	0.4	1.7–3.2	< 0.001
Appetite	Day 0	5.3 ± 1.5	–	–	–	–
	Day 14	7.1 ± 1.3	1.8	0.3	1.2–2.5	< 0.001
	Day 28	8.4 ± 1.7	3.0	0.4	2.1–3.9	< 0.001
Body weight	Day 0	8.6 ± 3.9	–	–	–	–
	Day 14	8.7 ± 3.9	0.1	0.0	0.0–0.2	0.217
	Day 28	8.4 ± 4.0	0.0	0.1	–0.1–0.2	0.695
Rectal temperature	Day 0	38.0 ± 0.7	–	–	–	–
	Day 14	38.0 ± 0.6	0.0	0.1	–0.1–0.1	0.812
	Day 28	38.1 ± 0.5	0.2	0.1	–0.1–0.4	0.163

*MD* Mean difference from Day 0, *SE* Standard error, *CI* Confidence interval. Sample sizes: Day 0 (*n* = 20), Day 14 (*n* = 20), Day 28 (*n* = 19)

### Effect of eqPE supplementation on haematological and biochemical parameters

No consistent treatment-related changes were observed in haematological or biochemical parameters during the study period, although individual variability was noted (Tables 3 and 4).

**Table 3:** Hematology of the dogs in supplementation between Day 0 and Day 28

Variable	Reference value	Day 0	Day 28
RBC (x10^6^/µL)	5.65–8.87	6.98 ± 1.18	7.20 ± 0.94
Ht (%)	37.3–61.7	44.7 ± 8.14	45.4 ± 5.50
Hb (g/dL)	13.1–20.5	15.6 ± 2.58	16.0 ± 1.95
MCV (fL)	61.6–73.5	64.0 ± 3.30	63.2 ± 2.99
MCH (pg)	21.2–25.9	22.3 ± 0.97	22.3 ± 1.10
MCHC (g/dL)	32.0–37.9	34.9 ± 1.65	35.3 ± 0.64
WBC (x 10^3^/µL)	5.05–16.76	9.05 ± 2.70	9.32 ± 3.85
Neu (x 10^3^/µL)	2.95–11.64	5.53 ± 2.29	5.70 ± 3.13
Lym (x 10^3^/µL)	1.05–5.10	2.32 ± 0.91	2.32 ± 0.92
Mon (x 10^3^/µL)	0.16–1.12	0.63 ± 0.22	0.68 ± 0.30
Eos (x 10^3^/µL)	0.06–1.23	0.53 ± 0.38	0.70 ± 0.80
Baso (x 10^3^/µL)	0.00–0.10	0.04 ± 0.07	0.02 ± 0.02
Plt (x 10^3^/µL)	148.0–484.0	230.0 ± 135.0	265.0 ± 140.0

Data are given as mean ± standard deviation (SD). *RBC* Red blood cells, *Ht* Haematocrit, *Hb* Haemoglobin, *MCV* Mean Corpuscular Volume, *MCH* Mean Corpuscular Hemoglobin, *MCHC* Mean Corpuscular Hemoglobin Concentration, *WBC* White blood cells, *Neu* Neutrophils, *Lym* Lymphocytes, *Mon* Monocytes, *Eos* Eosinophils, *Baso* Basophils, *Plt* Platelets. Sample sizes: Day 0 (*n* = 20), Day 28 (*n* = 19)

**Table 4:** Biochemistry of the dogs in supplementation between day 0 and day 28

Variable	Reference value	Day 0	Day 28
GLU (mg/dL)	70.0–143,0	80.0 ± 26.0	82.0 ± 30.0
CREA (mg/dL)	0.5–1.8	0.8 ± 0.3	0.8 ± 0.3
BUN (mg/dL)	7.0–27.0	18.0 ± 9.0	19.0 ± 7.0
BUN/CREA	–	23.0 ± 8.0	24.0 ± 7.0
PHOS (mg/dL)	2.5–6.8	4.1 ± 0.7	3.8 ± 0.6
Ca (mg/dL)	7.9–12.0	8.5 ± 2.7	7.8 ± 3.2
TP (g/dL)	5.2–8.2	6.9 ± 1.0	6.8 ± 0.7
ALB (g/dL)	2.2–3.9	3.2 ± 0.5	3.2 ± 0.4
GLOB (g/dL)	2.5–4.5	3.6 ± 0.6	3.6 ± 0.5
ALB/GLOB	–	0.9 ± 0.2	0.9 ± 0.2
ALT (U/L)	10.0–125.0	88.0 ± 47.0	80.0 ± 32.0
ALKP (U/L)	23.0–212.0	98.0 ± 261.0	111.0 ± 316.0
GGT (U/L)	0.0–11.0	4.0 ± 4.0	3.0 ± 4.0
TBIL (mg/dL)	0.0–0.9	0.4 ± 0.5	0.5 ± 0.5
CHOL (mg/dL)	110.0–320.0	177.0 ± 41.0	188.0 ± 39.0
Na (mmol/L)	144.0–160.0	152.0 ± 3.0	151.0 ± 4.0
K (mmol/L)	3.5–5.8	5.2 ± 0.5	5.0 ± 0.5
Na/K	–	30.0 ± 3.0	30.0 ± 3.0
Cl (mmol/L)	109.0–122.0	113.0 ± 5.0	110.0 ± 4.0
AMYL (U/L)	500.0–1500.0	703.0 ± 212.0	799.0 ± 437.0
LIPA (U/L)	200.0–1800.0	669.0 ± 425.0	632.0 ± 387.0
SDMA (µg/dL)	0.0–14.0	11.0 ± 5.0	10.0 ± 2.0
TT4 (µg/dL)	1.0–4.0	1.7 ± 0.9	1.9 ± 1.0
CRP (mg/dL)	0.0–1.0	0.7 ± 0.6	0.8 ± 0.6

Data are given as mean ± standard deviation (SD). *GLU* Glucose, *CREA* Creatinine, *BUN* Blood urea nitrogen, *PHOS* Phosphorus, *Ca* Calcium, *TP* Total protein, *ALB* Albumin, *GLOB* Globulin, *ALT* Alanine aminotransferase, *ALKP* Alkaline phosphatase, *GGT* Gamma-glutamyl transferase, *TBIL* Total bilirubin, *CHOL* Cholesterol, *Na* Sodium, *K* Potassium, *Cl* Chloride, *AMYL* Amylase, *LIPA* Lipase, *SDMA* Symmetric dimethylarginine, *TT4 *Total thyroxine, *CRP* C-reactive protein. Sample sizes: Day 0 (*n* = 20), Day 28 (*n* = 19)

### Adverse events during the study period

Adverse events were observed in two cases. One dog exhibited haematuria prior to initiation of eqPE administration and was therefore considered a pre-existing condition rather than an adverse event related to the intervention. A second dog developed severe pulmonary and intra-abdominal haemorrhage and died on Day 22. This event was suspected to be related to accidental rodenticide ingestion based on the owner’s statement and external veterinary reports; however, definitive confirmation was not available. The fatal event was considered unlikely to be related to the intervention.

### Additional observations

Exploratory physical activity data were collected; however, no statistical analysis was performed due to the small sample size (Additional file 1, 2, 3, and 4).

## Discussion

In this exploratory pre–post study in geriatric dogs with no control group, continuous oral administration of eqPE for 28 days was associated with improvements in ageing-level assessment scores, vitality, and appetite compared with those at baseline. To the best of our knowledge, this study is among the few to evaluate the potential functional effects of placental extract supplementation in elderly dogs exhibiting age-related declines.

Peptides derived from placental hydrolysates have demonstrated antioxidant activity [16], and similar antioxidative peptides have been characterised in sheep placental extracts following digestion [20]. Given the established antioxidative and anti-inflammatory properties of placental extracts [14], these reports support the hypothesis that small peptides or other constituents in eqPE may modulate cellular redox balance or mitochondrial function, thereby contributing to enhanced vitality, appetite, and overall ageing-related status in geriatric dogs. However, eqPE is a complex biological extract with undefined active components, and its mechanisms of action remain unclear.

In the present study, the observed improvements in the ageing-level assessment scores—a composite index reflecting coat condition, appetite, and behaviour—indicated that eqPE may mitigate certain aspects of age-associated physiological decline. The observed improvements in vitality and appetite may be influenced by factors other than ageing processes, including potential anti-inflammatory effects and improved palatability of the supplement. Ageing in dogs is commonly accompanied by increased oxidative stress, mitochondrial dysfunction, appetite dysregulation, and inflammation, all of which contribute to reduced quality of life [21, 22]. Recent multi-omics studies have further characterised these molecular alterations in canine ageing [23]. In this context, interventions that safely improve vitality and appetite without inducing adverse effects may offer supportive benefits in geriatric veterinary care.

No significant changes in body weight or rectal temperature were observed during the study period. The absence of significant changes in body weight may reflect the short duration of the study and inter-individual variability. Similarly, no consistent treatment-related changes were observed in haematological and biochemical parameters during the study period, although individual variability was noted. The single fatal event was considered unlikely to be related to the intervention.

Future studies with larger sample sizes and continuous activity monitoring are warranted to further evaluate the effects of eqPE supplementation on physical activity.

The present study is subject to several limitations. A major limitation of this study is the absence of a control group, which precludes causal inference and introduces a high likelihood of placebo effects; therefore, the findings should be interpreted with caution. Firstly, it was exploratory in nature and utilized a single-arm pre–post design. This study was neither controlled nor blinded, and thus the observed improvements may have been strongly influenced by factors such as placebo effects, the natural course of age-related decline, or observer/owner bias. Secondly, the small sample size and relatively short 28-day follow-up period restricted the evaluation of long-term efficacy and the detection of rare adverse events. Thirdly, given that eqPE is a complex biological extract with undefined active components and mechanisms of action, causal interpretation was limited. The safety evaluation was limited to haematological parameters and liver- and kidney-function markers and did not constitute a comprehensive toxicological assessment. Consequently, although the present findings are encouraging, further validation through randomised, placebo-controlled trials with longer follow-up periods, standardised formulations, and mechanistic investigations—including biomarker and pharmacological analyses—is required.

## Conclusions

In this exploratory single-group pre–post study, 28 days of oral eqPE administration was associated with improvements in ageing-level assessment scores, vitality, and appetite in geriatric dogs. No apparent abnormalities were detected in haematological or biochemical parameters. These findings suggest that eqPE may have potential as an adjunctive intervention for age-related declines in vitality and appetite; however, these findings are preliminary and require confirmation in controlled studies. Given the limitations of the single-group design, small sample size, and short observation period, these results should be considered preliminary. Further studies should incorporate larger, randomised controlled trials and include efforts to elucidate the underlying mechanisms of action.

## Methods

### Study animals and design

This study was conducted and reported in accordance with the ARRIVE guidelines, where applicable. The completed ARRIVE checklist is provided as Additional file 5. The primary objective of this study was to obtain preliminary data to support the design of future controlled trials. The study was conducted at veterinary hospitals in Maebashi City (Gunma Prefecture), Ueda City (Nagano Prefecture), Kumamoto City (Kumamoto Prefecture), and Haebaru Town (Okinawa Prefecture), Japan, between October 2024 and May 2025. The protocol was approved by the Shokukanken Inc. Animal Experimentation Committee (Approval Number: AW24Jul002O), and written informed consent was obtained from all dog owners prior to enrolment. Study conduct was overseen by a commissioned research organisation (Shokukanken Inc., Gunma, Japan).

All dogs were client-owned and maintained under their owners’ usual home management conditions throughout the study. No experimental housing or husbandry modifications were imposed. The intervention consisted solely of oral administration of a dietary supplement, and no invasive procedures were performed. Animal welfare was monitored by attending veterinarians during scheduled visits and throughout the study period, and appropriate clinical care was provided if any signs of pain, suffering, or distress were observed.

Client-owned dogs aged 11 years or older were eligible for inclusion regardless of breed or sex. Animals were included if they exhibited at least one of the following age-associated clinical signs: deterioration of coat or skin, reduced appetite, and/or decreased physical activity level. Given the exploratory nature of this single-group pre–post study, no strict predefined exclusion criteria were applied; however, dogs presenting with clinically significant abnormalities (e.g., haematuria) were evaluated at the discretion of the attending veterinarian and managed appropriately. However, dogs deemed unsuitable by the attending veterinarian, owing to severe disease or poor general condition, were not enrolled. Randomisation was not performed because this study was designed as an exploratory, single-group pre–post study without a comparator. Blinding was not performed because this study was a single-group pre–post study in which all dogs received the same intervention, and no group allocation was involved.

No euthanasia was performed as part of this study. Humane endpoints were predefined to minimise animal pain, suffering, and distress. Dogs were withdrawn from the study if any of the following conditions occurred: (1) the need for major surgery during the study period; (2) the development of diseases that could affect study outcomes; or (3) the occurrence of adverse events that interfered with the objectives of the study (for example, inability to continue assessments due to trauma such as a traffic accident, or infectious diseases causing persistent vomiting, diarrhoea, or severe weight loss). All decisions regarding study withdrawal were made by the attending veterinarian in the best interest of animal welfare.

**Fig. 2:**
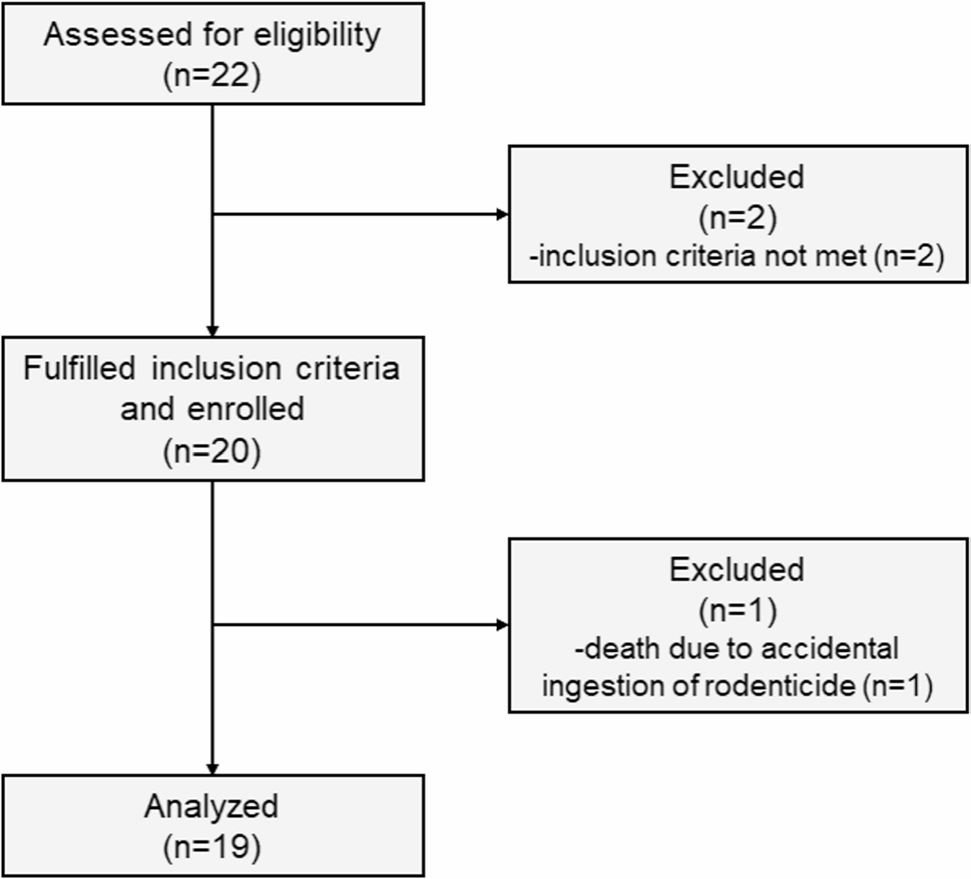
Flowchart showing selection of participants for equine placental extract supplementation study. The diagram illustrates assessment of eligibility of dogs to receive equine placental extract supplementation, application of exclusion criteria, enrolment of participants, and inclusion in the final analyses

A total of 22 dogs were assessed for eligibility. Of the 22 dogs assessed for eligibility, two dogs were not enrolled because, although they met the age criterion, they did not exhibit any of the predefined age-associated clinical signs required for inclusion. Of the 20 enrolled dogs, one dog ingested a rodenticide on Day 20 and died on Day 22 due to severe pulmonary and intra-abdominal haemorrhage, preventing completion of the protocol. This case was excluded from the Day 28 analysis; whereas its data were retained for Day 14 efficacy and safety analyses. The study concluded with 20 dogs completing participation (Fig. 2). The sample size was based on feasibility and budget constraints, rather than statistical power.

Baseline characteristics of the dogs before supplementation are summarized in Table 5. The median age of the dogs was 11.5 years (range: 11.0–18.0 years). The median weight of the dogs was 9.3 kg (range: 2.6–15.1 kg). The population comprised 10 male dogs (7 neutered) and 10 female dogs (2 spayed). Six dogs were of mixed-breed, and 14 belonged to four distinct breeds.

**Table 5:** Baseline characteristics of the dogs before supplementation

Characteristics	Dogs used in the study (*n* = 20)
Age (years)	11.5 (11.0–18.0)
Weight (kg)	9.3 (2.6–15.1)
Sex (number, %)	
Male Female	10 (50) 10 (50)
Spayed/neutered (number, %)	7 (35)
Breed (number)	
Toy Poodle Shiba Inu Beagle Mixed Poodle	1 7 1 6 5
Aging score	22.9 ± 9.2

The age and weight of the subjects were shown as median (range) and number (percentage), respectively

### Study product and intervention protocol

The study product was eqPE (Japan Bio Products Co., Ltd., Tokyo, Japan), a placental extract produced by enzymatic hydrolysis of horse placenta according to an internal manufacturing protocol (internal data). For the intervention, eqPE was administered orally once daily for 28 consecutive days. The dosage was adjusted according to body weight—1 mL for dogs weighing ≤ 5.0 kg and 2 mL for dogs weighing 5.1–19.9 kg. The supplement was administered either directly into the mouth or mixed with feed. Compliance with eqPE administration was assessed using daily logs completed by the owners.

### Study procedures and data collection

The schedule of study assessment is summarized in Table 6. Assessments of the general condition of the dogs were conducted at screening (Day − 7), the start of eqPE administration (Day 0; baseline), and post-administration (Days 14 and 28). Assessments included interviews with owners and physical examinations. Scoring was performed according to the canine ageing-level assessment score (Japan Pet Supplement Association, Simplified Health Check Sheet Vol. 2) (Additional file 6). This composite score incorporates multiple indicators, including behaviour, appetite, and coat condition. As the original scale was available only in Japanese, an authorised English translation of the assessment tool was used, with formal authorization from the copyright holder, and is provided in the Supplementary Materials (Additional file 6). Vitality and appetite were assessed using VASs, each ranging from 0 to 10, with higher scores indicating better vitality or appetite. Haematological and biochemical analyses were conducted at baseline (Day 0) and at the end of the intervention (Day 28). Adverse events were monitored throughout the study period.

**Table 6:** Schedule of events

Event	Day − 7	Day 0	Day 14	Day 28
Screen for inclusion criteria	✓			
Obtain owner informed consent	✓			
Review inclusion criteria		✓		
Equine placenta extract supplementation		✓	✓	✓
Observation of general condition	✓	✓	✓	✓
Questionnaire for aging level	✓	✓	✓	✓
Hematology and biochemistry		✓		✓

In addition, physical activity data were collected using PLUS CYCLE^®^ devices for exploratory purposes [24]; however, these data were not included in the primary statistical analyses due to the small sample size.

### Monitoring of adverse events

An adverse event was defined as any unfavourable or unintended clinical sign observed during the study period, irrespective of its causal relationship to the study product. Serious adverse events were defined as events resulting in death, life-threatening conditions, permanent disability, and/or a need for intensive or prolonged veterinary care. All abnormal clinical signs observed after initiation of the intervention were systematically recorded as adverse events. Pre-existing conditions were not classified as adverse events unless worsening was observed after study initiation. Furthermore, adverse events recurring at consecutive time points were recorded as a single event, unless they reappeared after resolution.

### Statistical analyses

Canine ageing-level assessment scores were expressed as median (IQR), whereas clinical variables were expressed as mean ± standard deviation with 95% CI. Statistical analyses were performed using IBM SPSS Statistics version 23 (IBM Japan Ltd., Tokyo, Japan) by independent statisticians at ORTHOMEDICO Inc. (Japan). Changes in canine-ageing level assessment scores were analysed using the Wilcoxon signed-rank test with Bonferroni correction, and clinical variable values were analysed using paired *t*-tests. Statistical significance was set at *p* < 0.05.

## Supplementary Information

Additional file 1: Fig. S1.

Additional file 2: Fig. S2.

Additional file 3: Fig. S3.

Additional file 4: Fig. S4.

Additional file 5: Table S1.

Additional file 6: Table S2.

## Data Availability

The datasets supporting the conclusions of this article are included within the article (and its additional file(s)).

## Abbreviations

CI: Confidence interval
eqPE: Equine placental extract
IQR: Interquartile range
PLE: Protein-losing enteropathy
VAS: Visual analogue scale
