# Supplementary figures and images for: Exploratory evaluation of equine placental extract supplementation on ageing indicators in geriatric dogs: a single-arm pre–post study
Source: BMC Vet Res. 2026 Apr 15;22:311. doi: 10.1186/s12917-026-05475-y (PMC13202951; doi:10.1186/s12917-026-05475-y)

## Slide 1
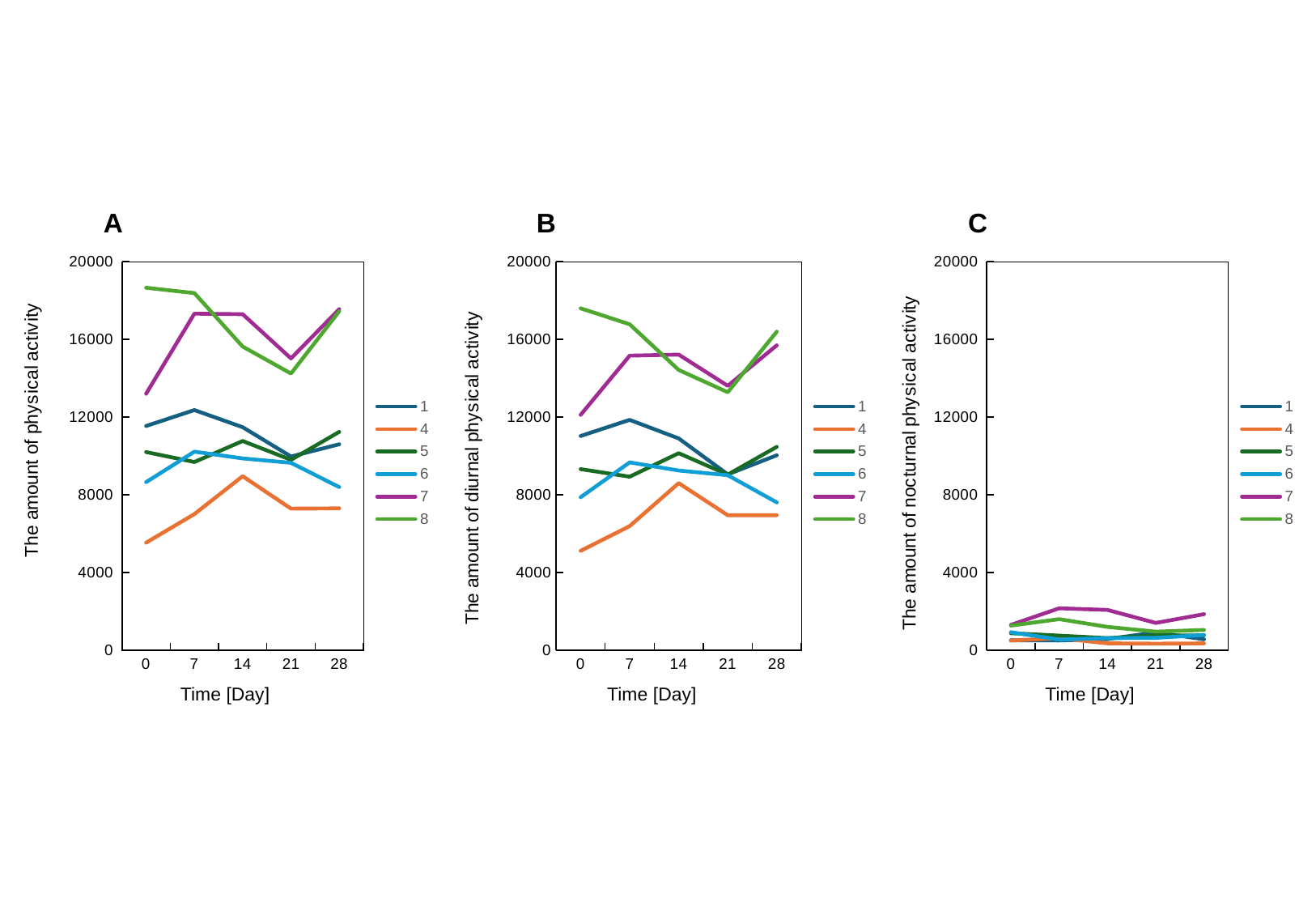

A
B
C
### Chart
| Category | 1 | 4 | 5 | 6 | 7 | 8 |
|---|---|---|---|---|---|---|
| 0 | 11543.0 | 5537.0 | 10203.0 | 8652.0 | 13203.0 | 18658.0 |
| 7 | 12363.0 | 7010.0 | 9690.0 | 10222.0 | 17325.0 | 18381.0 |
| 14 | 11483.0 | 8959.0 | 10771.0 | 9876.0 | 17297.0 | 15632.0 |
| 21 | 9973.0 | 7290.0 | 9801.0 | 9649.0 | 15021.0 | 14243.0 |
| 28 | 10605.0 | 7304.0 | 11242.0 | 8396.0 | 17555.0 | 17446.0 |
### Chart
| Category | 1 | 4 | 5 | 6 | 7 | 8 |
|---|---|---|---|---|---|---|
| 0 | 11030.0 | 5117.0 | 9323.0 | 7876.0 | 12114.0 | 17600.0 |
| 7 | 11859.0 | 6389.0 | 8934.0 | 9671.0 | 15164.0 | 16779.0 |
| 14 | 10905.0 | 8600.0 | 10145.0 | 9250.0 | 15218.0 | 14428.0 |
| 21 | 9052.0 | 6950.0 | 9040.0 | 9010.0 | 13611.0 | 13282.0 |
| 28 | 10038.0 | 6949.0 | 10471.0 | 7610.0 | 15695.0 | 16398.0 |
### Chart
| Category | 1 | 4 | 5 | 6 | 7 | 8 |
|---|---|---|---|---|---|---|
| 0 | 513.0 | 503.0 | 880.0 | 931.0 | 1306.0 | 1270.0 |
| 7 | 505.0 | 621.0 | 756.0 | 551.0 | 2161.0 | 1602.0 |
| 14 | 578.0 | 359.0 | 627.0 | 626.0 | 2079.0 | 1204.0 |
| 21 | 921.0 | 340.0 | 761.0 | 640.0 | 1409.0 | 961.0 |
| 28 | 567.0 | 355.0 | 771.0 | 786.0 | 1860.0 | 1048.0 |Time [Day]
Time [Day]
Time [Day]

Supplement: Supplementary file 1 — Additional file 1: Fig. S1. [file 12917_2026_5475_MOESM1_ESM.pptx]

## Slide 1
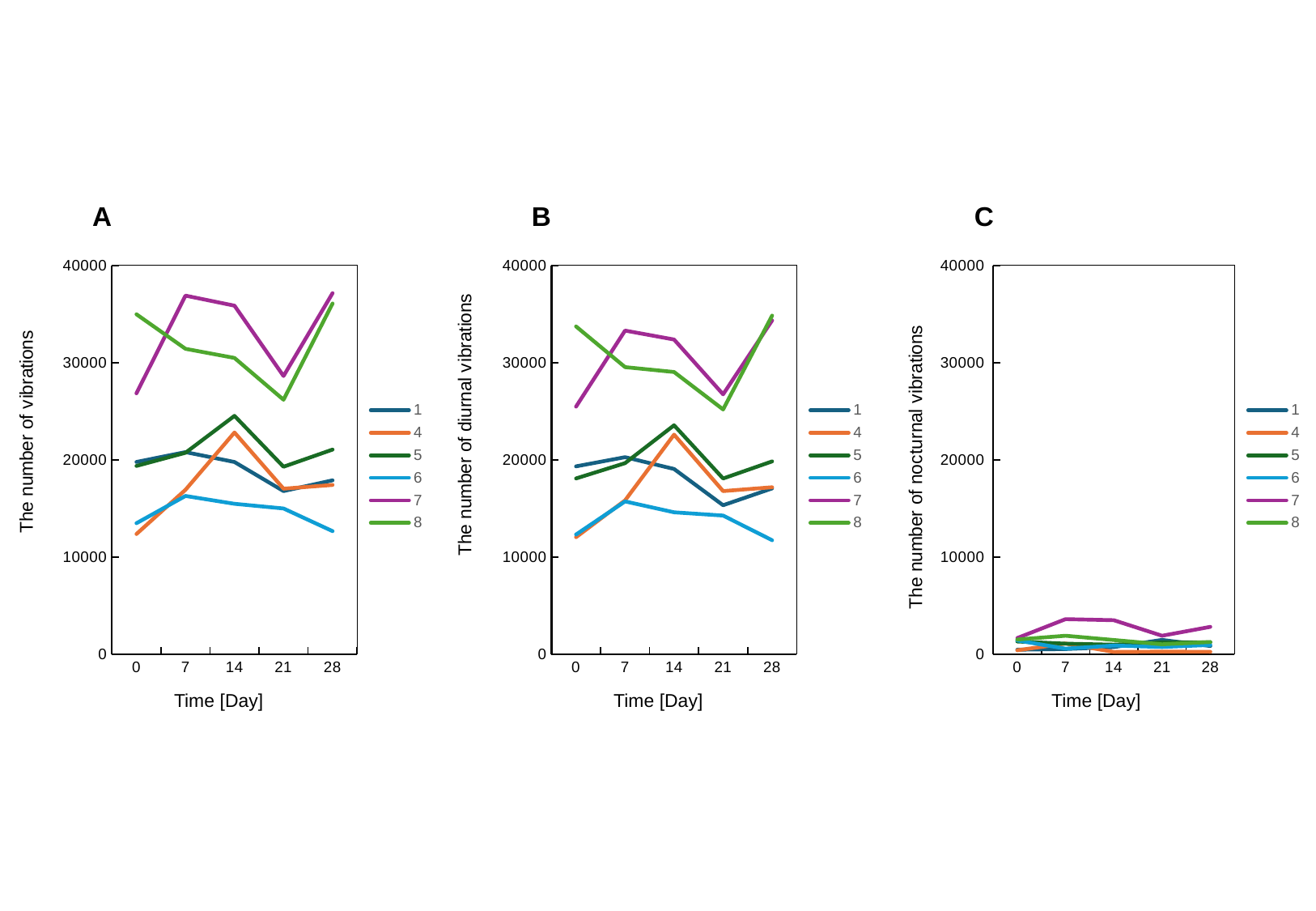

A
B
C
### Chart
| Category | 1 | 4 | 5 | 6 | 7 | 8 |
|---|---|---|---|---|---|---|
| 0 | 19775.0 | 12356.0 | 19370.0 | 13468.0 | 26842.0 | 34971.0 |
| 7 | 20788.0 | 16905.0 | 20722.0 | 16270.0 | 36890.0 | 31421.0 |
| 14 | 19769.0 | 22807.0 | 24520.0 | 15466.0 | 35860.0 | 30482.0 |
| 21 | 16775.0 | 17022.0 | 19277.0 | 14981.0 | 28619.0 | 26181.0 |
| 28 | 17882.0 | 17404.0 | 21052.0 | 12652.0 | 37150.0 | 36083.0 |
### Chart
| Category | 1 | 4 | 5 | 6 | 7 | 8 |
|---|---|---|---|---|---|---|
| 0 | 19323.0 | 12031.0 | 18072.0 | 12299.0 | 25468.0 | 33722.0 |
| 7 | 20266.0 | 15820.0 | 19651.0 | 15710.0 | 33294.0 | 29535.0 |
| 14 | 19048.0 | 22586.0 | 23553.0 | 14587.0 | 32374.0 | 29032.0 |
| 21 | 15317.0 | 16774.0 | 18067.0 | 14252.0 | 26733.0 | 25178.0 |
| 28 | 17051.0 | 17170.0 | 19835.0 | 11716.0 | 34345.0 | 34842.0 |
### Chart
| Category | 1 | 4 | 5 | 6 | 7 | 8 |
|---|---|---|---|---|---|---|
| 0 | 452.0 | 390.0 | 1298.0 | 1403.0 | 1649.0 | 1498.0 |
| 7 | 521.0 | 1085.0 | 1071.0 | 560.0 | 3596.0 | 1886.0 |
| 14 | 721.0 | 221.0 | 967.0 | 880.0 | 3485.0 | 1451.0 |
| 21 | 1458.0 | 249.0 | 1210.0 | 729.0 | 1886.0 | 1004.0 |
| 28 | 831.0 | 234.0 | 1217.0 | 937.0 | 2805.0 | 1242.0 |Time [Day]
Time [Day]
Time [Day]

Supplement: Supplementary file 2 — Additional file 2: Fig. S2. [file 12917_2026_5475_MOESM2_ESM.pptx]

## Slide 1
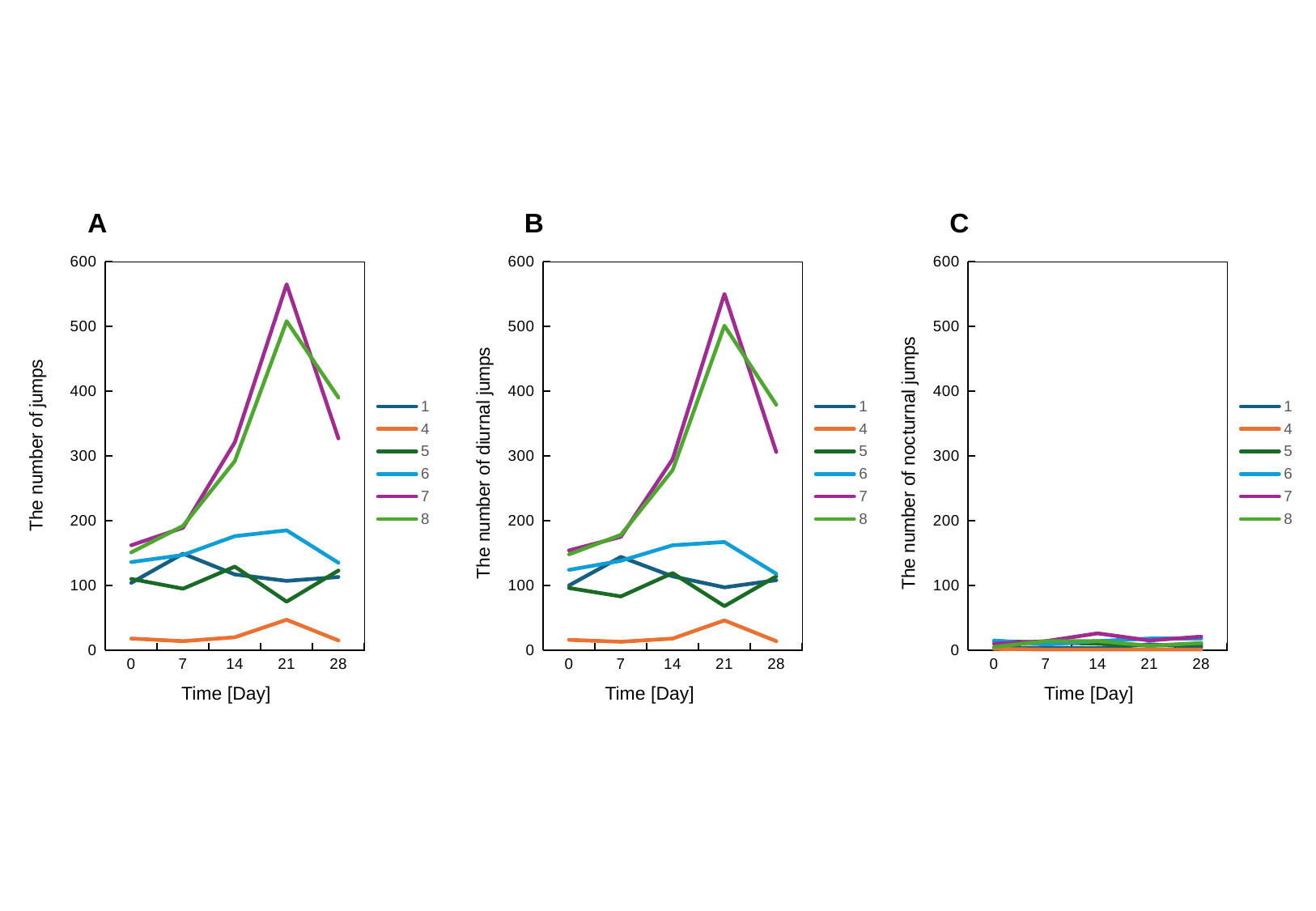

A
B
C
### Chart
| Category | 1 | 4 | 5 | 6 | 7 | 8 |
|---|---|---|---|---|---|---|
| 0 | 104.0 | 18.0 | 110.0 | 136.0 | 162.0 | 151.0 |
| 7 | 149.0 | 14.0 | 95.0 | 147.0 | 189.0 | 192.0 |
| 14 | 117.0 | 20.0 | 129.0 | 176.0 | 321.0 | 292.0 |
| 21 | 107.0 | 47.0 | 75.0 | 185.0 | 565.0 | 508.0 |
| 28 | 113.0 | 15.0 | 123.0 | 135.0 | 327.0 | 390.0 |
### Chart
| Category | 1 | 4 | 5 | 6 | 7 | 8 |
|---|---|---|---|---|---|---|
| 0 | 100.0 | 16.0 | 96.0 | 124.0 | 154.0 | 148.0 |
| 7 | 144.0 | 13.0 | 83.0 | 138.0 | 175.0 | 178.0 |
| 14 | 114.0 | 18.0 | 119.0 | 162.0 | 295.0 | 278.0 |
| 21 | 97.0 | 46.0 | 68.0 | 167.0 | 550.0 | 501.0 |
| 28 | 108.0 | 14.0 | 114.0 | 118.0 | 306.0 | 379.0 |
### Chart
| Category | 1 | 4 | 5 | 6 | 7 | 8 |
|---|---|---|---|---|---|---|
| 0 | 3.0 | 2.0 | 14.0 | 15.0 | 10.0 | 5.0 |
| 7 | 4.0 | 1.0 | 12.0 | 9.0 | 14.0 | 14.0 |
| 14 | 3.0 | 1.0 | 10.0 | 14.0 | 26.0 | 14.0 |
| 21 | 9.0 | 1.0 | 7.0 | 18.0 | 15.0 | 7.0 |
| 28 | 5.0 | 1.0 | 9.0 | 18.0 | 21.0 | 11.0 |Time [Day]
Time [Day]
Time [Day]

Supplement: Supplementary file 3 — Additional file 3: Fig. S3. [file 12917_2026_5475_MOESM3_ESM.pptx]

## Slide 1
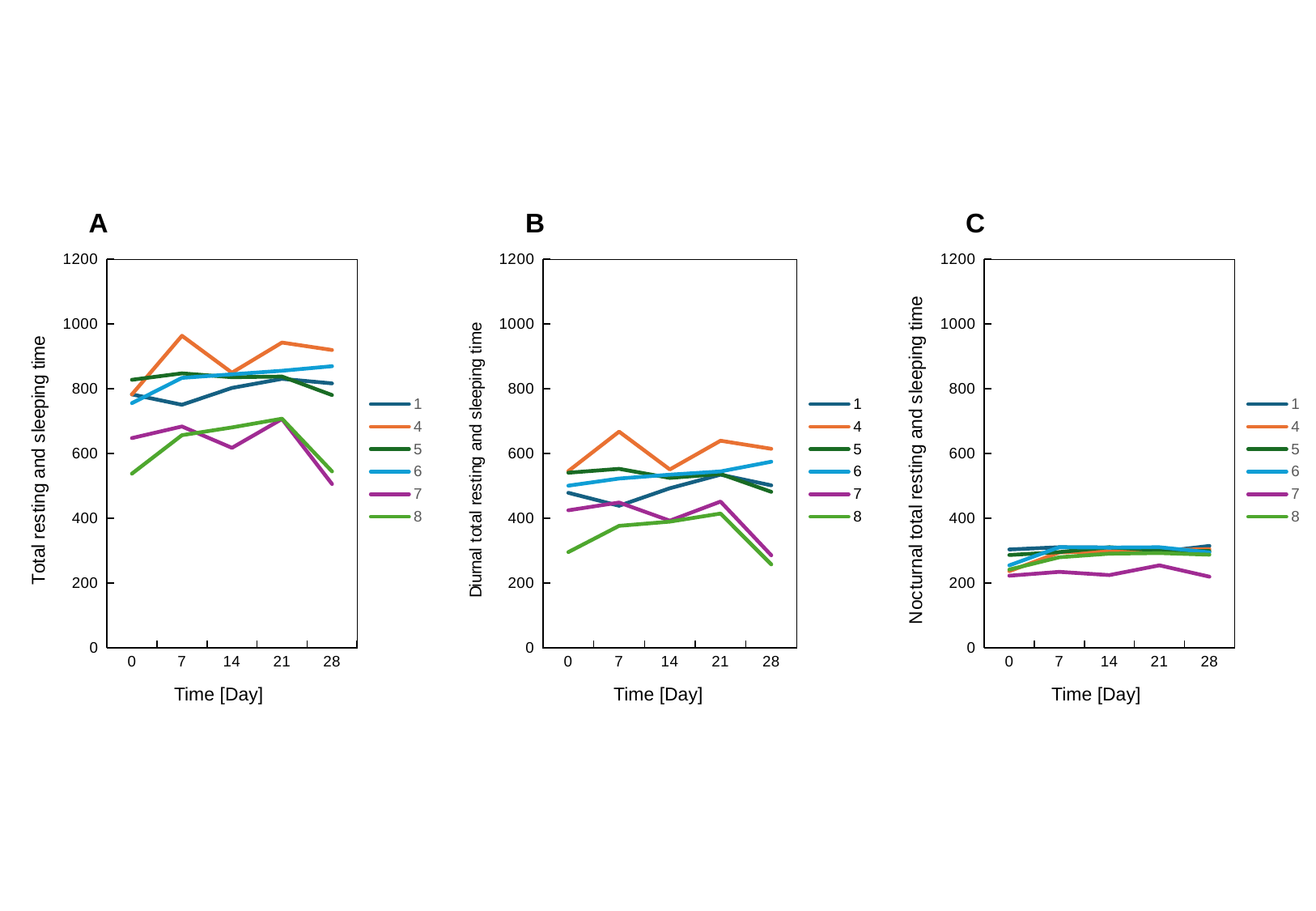

A
B
C
### Chart
| Category | 1 | 4 | 5 | 6 | 7 | 8 |
|---|---|---|---|---|---|---|
| 0 | 783.0 | 783.0 | 828.0 | 756.0 | 648.0 | 538.0 |
| 7 | 751.0 | 964.0 | 848.0 | 834.0 | 684.0 | 657.0 |
| 14 | 803.0 | 850.0 | 836.0 | 845.0 | 618.0 | 681.0 |
| 21 | 831.0 | 943.0 | 838.0 | 856.0 | 707.0 | 708.0 |
| 28 | 817.0 | 920.0 | 781.0 | 870.0 | 506.0 | 545.0 |
### Chart
| Category | 1 | 4 | 5 | 6 | 7 | 8 |
|---|---|---|---|---|---|---|
| 0 | 479.0 | 546.0 | 541.0 | 501.0 | 425.0 | 296.0 |
| 7 | 439.0 | 668.0 | 553.0 | 523.0 | 449.0 | 377.0 |
| 14 | 493.0 | 551.0 | 525.0 | 535.0 | 393.0 | 390.0 |
| 21 | 535.0 | 640.0 | 537.0 | 545.0 | 452.0 | 415.0 |
| 28 | 502.0 | 615.0 | 482.0 | 575.0 | 286.0 | 258.0 |
### Chart
| Category | 1 | 4 | 5 | 6 | 7 | 8 |
|---|---|---|---|---|---|---|
| 0 | 304.0 | 237.0 | 287.0 | 255.0 | 223.0 | 242.0 |
| 7 | 311.0 | 295.0 | 296.0 | 311.0 | 235.0 | 280.0 |
| 14 | 310.0 | 298.0 | 311.0 | 310.0 | 225.0 | 291.0 |
| 21 | 297.0 | 302.0 | 301.0 | 311.0 | 255.0 | 293.0 |
| 28 | 315.0 | 305.0 | 299.0 | 295.0 | 220.0 | 288.0 |Time [Day]
Time [Day]
Time [Day]

Supplement: Supplementary file 4 — Additional file 4: Fig. S4. [file 12917_2026_5475_MOESM4_ESM.pptx]
