# Supplementary material for: Exploratory evaluation of equine placental extract supplementation on ageing indicators in geriatric dogs: a single-arm pre–post study
Source: BMC Vet Res. 2026 Apr 15;22:311. doi: 10.1186/s12917-026-05475-y (PMC13202951; doi:10.1186/s12917-026-05475-y)
Supplement: Supplementary file 6 — Additional file 6: Table S2. [file 12917_2026_5475_MOESM6_ESM.docx]

**Table S1** **Questionnaire of Simplified Health Check Table, Vol. 2 (Japan Pet Supplement Association)**

**Physical Examination Information (Date: XX/YY/20ZZ)**

[Day 7, Day 0, Day 14 ± 3, Day 28 ± 3]

◎Please draw (an illustration) any areas of concern on the dog.

First, gently touch the entire body of your dog.

Next, check for any unpleasant odors or secretions in the eyes, ears, nose, mouth, and body.

Observe any changes in the coat luster or skin color.

Carefully examine the underside of the pads and around the anus, without causing any discomfort.

Thoroughly check the entire body and make a note or draw (an illustration) any areas of concern.


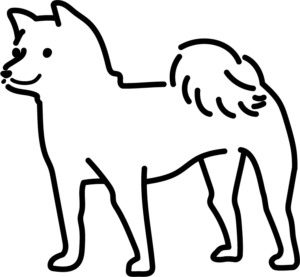


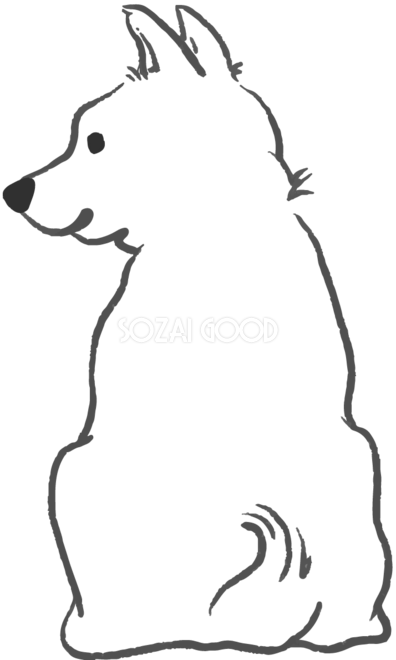


―――――――――――――――――――――――――――――――――――――

Finally, if you have any comments or suggestions, please feel free to share them.

**Aging Level Assessment Score (1) (Date: XX/YY/20ZZ)**

**I. Body**

1. 1. Have you noticed any unusual changes in the physical condition of your dog over the past few days or the past month? (Select only one)

0p: □ Nothing unusual. In fact, it appears to feel better.

1p: □ Nothing unusual, and its physical health condition has remained the same for the past few years.

2p: □ It appears older with age, but I do not notice any changes in its physical condition.

3p: □ It occasionally gets sick but always gets better.

4p: □ This is the worst physical condition I have ever seen.

*If you noticed any changes, please describe the condition, and if known, please also describe the reason.

Body Areas, Symptoms, and Cause:

1. How strong is its body odor? (Select only one)

0p: □ Not at all bothersome.

1p: □ It is only noticeable under certain weather conditions, such as when it gets wet on a rainy day.

2p: □ The odor is sometimes noticeable even after wiping.

3p: □ The odor is noticeable if I do not wash it for a long time.

4p: □ The odor is noticeable even after washing it, and people outside of my family have pointed it out to me.

*If you know the extent or cause of its body odor, please describe it.

Reason:

1. Has your dog lost or gained weight within a short period of time (weeks to months)? (Select only one)

0p: □ Its appearance and weight have not changed.

1p: □ Its appearance has not changed in 1–3 months, but it has gained or lost weight over the 1–3 months period.

2p: □ Its appearance has not changed, but it has gained or lost weight within approximately 1 month.

3p: □ Its appearance and weight have both increased or decreased within 1 month.

4p: □ Its weight has increased or decreased dramatically within a few weeks.

*If you know the cause of its sudden weight loss or gain, please describe it.

Reason (previous weight vs. current weight, etc.):

1. Does any of the following apply to the overall appearance of your dog? (0 or multiple answers allowed)

1p: □ The jaw and mouth line have sagged, and the muscles in the face and neck seem to be weakening.

1p: □ Pigmentation and blemishes are on the skin areas with thin fur.

1p: □ Decreased saliva production and bad breath due to conditions such as periodontal disease.

1p: □ Presence of a lump or other tumor in a certain part of the body.

1p: □ The back seems rounded, and the spine appears to have sagged.

*If you know the cause, please describe it.

Reason:

**Aging Level Assessment Score (2) (Date: XX/YY/20ZZ)**

**II. Hair (coat)**

1. Is there anything abnormal with the coat? (Select only one)

0p: □ Shiny and healthy hair, with no hair loss. The coat retains its luster as it did when the dog was 2 or 3 years old.

1p: □ Not very shiny, and abnormalities such as dandruff are visible depending on the season.

2p: □ Dull and coarse coat, with slow growth rate.

3p: □ The coat appears matte, with areas of coarseness, hairballs, hair loss, and noticeable gray hairs.

4p: □ Overall coarseness, hairballs, and hair loss are present. The entire face appears pale, with noticeable fading of pigment.

*If there are any abnormalities, please describe the affected body areas, symptoms, and cause, if known.

Body areas, Symptoms, and Cause:

**III. Skin**

1. Is there anything abnormal with the skin? (Select only one)

0p: □ The skin is completely normal, smooth, and healthy.

1p: □ Slightly dry, with seasonal abnormalities such as eczema.

2p: □ Loss of firmness. Scratching and claw marks are visible in some areas.

3p: □ Itching and blotches are present in thin areas of the skin, such as around the eyes and abdomen.

4p: □ The entire body is itchy, with widespread skin disease and fur damage, and many red, festering areas.

*If there are any abnormalities, please describe the affected body areas, symptoms, and cause, if known.

Body areas, Symptoms, and Cause:

**IV. Mouth and Tongue**

1. Have you noticed any abnormalities in the mouth or tongue? (Select only one)

0p: □ No breath odor. The gums and tongue are clean and unchanged from when it was 2–3 years old.

1p: □ Excessive saliva, constant drooling.

2p: □ Slightly dry, with difficulty in swallowing. A small amount of tartar is present.

3p: □ Noticeable dirt around the mouth and mild bad breath. A small amount of tartar is present.

4p: □ Breath has a strong stench, and the tongue is dry with a high amount of tartar present.

*If there are any abnormalities, please describe the condition in detail and provide the reason, if known.

Symptoms/Cause:

1. Have you noticed any breathing or stomach problems? (Select only one)

0p: □ No problems. Condition is unchanged from when it was 2–3 years old.

1p: □ Breathing is slow at rest, but heavier than before during activity.

2p: □ Whistling sound when breathing, both at rest and when active.

3p: □ Occasional coughing or vomiting of stomach acid, both at rest and when active.

4p: □ Coughing, tendency to hyperventilate, and frequent vomiting.

*If there are any abnormalities, please describe the condition in detail and provide the reason, if known.

Symptoms/Cause:

**Aging Level Assessment Score (3) (Date: XX/YY/20ZZ)**

**V. Eyes**

1. Are there any abnormalities around the eyes? (Select only one)

0p: □ No abnormalities around the eyes, and the fur is healthy and well-grown.

1p: □ Slight swelling around the eyes, with watery discharge due to tear stains.

2p: □ Redness around the eyes, and the eyes are mostly closed, making it difficult to see.

3p: □ Redness with festering around the eyes, along with itchiness and rubbing marks.

4p: □ The festering has spread to the entire face, with visible scratch marks.

*If there are any abnormalities, please describe the condition in detail and provide the reason, if known.

Symptoms/Cause:

1. Are there any issues with the vision or corneas? (Select only one)

0p: □ The corneas are clean and not cloudy at all. The eyes look healthy.

1p: □ Slight weakening of eyesight, but no interference with daily life.

2p: □ Does not follow objects with the eyes, or bumps into objects in the dark.

3p: □ Bumps into objects even during the day, suggesting severely impaired vision.

4p: □ The eyes are completely cloudy, with total loss of vision.

*If there are any issues, please describe the condition in detail and provide the reason, if known.

Reason/Cause:

**VI. Ears and Nose**

1. Are there any abnormalities in the ears or surrounding area? (Select only one)

0p: □ The inside of the ears is clean and fresh, with no external abnormalities. Responds normally to sounds.

1p: □ Occasionally touches or shakes its ears to remove foreign objects every now and then.

2p: □ Dirt is present inside the ears; the surrounding area is slightly dirty and has a mild odor.

3p: □ The inside of the ears has a foul odor and becomes dirty easily, even after cleaning. Responds poorly to sounds.

4p: □ The inside of the ears is festering, with widespread dirt and a strong odor. No response to sounds.

*If there are any abnormalities, please describe the condition in detail and provide the reason, if known.

Symptoms/Cause:

1. Are there any abnormalities in the nose or surrounding area?

0p: □ No apparent abnormalities; appetite is good and frequent sniffing occurs during walks.

1p: □ Occasionally rubs the nose against objects in an attempt to remove foreign matter.

2p: □ Nose appears slightly dry; frequent sniffing observed.

3p: □ Nose remains dry throughout the day, or colored nasal discharge is present.

4p: □ Nose is non-functional; no interest in smells is shown.

*If there are any abnormalities, please describe the condition in detail and provide the reason, if known.

Symptoms/Cause:

**Aging Level Assessment Score (4) (Date: XX/YY/20ZZ)**

**VII. Excrement**

1. What is the consistency of the stools of your dog? Please also indicate the number of times per day. (Select only one)

0p: □ Normal appearance; banana-shaped, healthy stools (water content approximately 70–80%)

1p: □ Small, round, rabbit-like stools with slight constipation (water content approximately 60%–70%)

2p: □ Hard, low-moisture stools that are difficult to pass (water content approximately 60% or less)

3p: □ Soft, unformed stools with excess moisture (water content approximately 80%–90%)

4p: □ Watery stools. Sometimes contains blood (water content approximately 90% or more)

*If there are any abnormalities, including the number of times per day, please provide the reason or cause, if known.

Times per day, amount, and length:

1. Which condition best describes the color of the stools of your dog? (Select only one)

0p: □ Yellowish-brown (yellowish-brown stools)

1p: □ Orange, reddish-brown, or brown (Reddish-brown stools)

2p: □ Bright red or green (blood-stained or dark green stools)

3p: □ Grayish-brown (cement- or lime-colored stools)

4p: □ Dark brown or tarry (pitch-black squid ink-colored stools)

*If known, please provide the reason for the abnormalities.

Cause:

1. Which condition best describes the odor of the stool of your dog? (Select only one)

0p: □ Little to no odor; not foul.

1p: □ Slight odor, not very bothersome.

2p: □ Noticeably strong odor.

3p: □ Presence of strong sour, acidic odor

4p: □ Presence of a putrefaction-like smell, and it reeks unbearably.

*If known, please provide the reason for the apparent abnormalities.

Reason:

1. What is the condition of your dog when using the toilet? (0 or multiple answers allowed)

1p: □ Shortened time between trips to the toilet.

1p: □ Increased frequency of accidents, despite previous proper toileting.

1p: □ Urinates small amounts repeatedly.

1p: □ Stool volume has decreased; difficulty in passing stools even when in toileting posture.

*If known, please provide the reason.

Reason:

**Aging Level Assessment Score (5) (Date: XX/YY/20ZZ)**

**VIII. Legs**

1. Do you notice any abnormalities in the legs and lower back? (Select only one)

0p: □ No issue with touching the legs or lower back; gait appears light and normal.

1p: □ No issue with touching the legs and lower back, but massages and other treatments raise some concerns.

2p: □ Discomfort when legs or lower back are touched; cannot climb steps without a run-up or assistance.

3p: □ Irritated when touched in these areas; unable to climb steps without assistance or being carried.

4p: □ Severe irritation when touched; unable to climb up or down even small steps.

*If you feel something is wrong, please describe the condition in detail and provide the reason, if known.

Areas, Symptoms, and Cause:

1. How is the walking performance of your dog? (0 or multiple answers allowed)

1p: □ Walking speed is slow.

1p: □ Walk appears listless and plodding.

1p: □ Often rests lying down.

1p: □ Reluctant to go up and down stairs.

1p: □ Sometimes drags its legs or cannot place them properly on the floor immediately after waking up.

*If known, please provide the reason.

Reason:

**IX. Spine**

1. Have you noticed any abnormalities in the condition of the spine of your dog? (Select only one)

0p: □ No stiffness or creasing felt when stroking the spine.

1p: □ Gentle stroking reveals stiffness, but none is felt when stroked more firmly .

2p: □ Firm stroking reveals stiffness, which subsides with massage.

3p: □ Its spine is inflexible, and it avoids going up and down slopes or stairs.

4p: □ It reacts painfully when its spine is touched, and bumps and lumps are present.

*If you feel something is wrong, please describe the condition in detail and provide the reason, if known.

Areas, Symptoms, and Cause:

**X. Energy Level**

1. 20. How energetic is your dog? (Select only one)

0p: □ Active, like a 2- or 3-year-old, the most energetic I have ever seen.

1p: □ Energetic and powerful, but quieter than before.

2p: □ Energetic but has calmed down for quite some time.

3p: □ Energy is weakening; signs of aging are apparent.

4p: □ Least energetic I’ve ever seen.

*If it is not energetic, please provide the reason, if known.

Reason:

**Aging Level Assessment Score (6) (Date: XX/YY/20ZZ)**

**XI. Daily Life**

1. Does your dog dislike walks? Please describe the total time spent walking and the quality of the surface. (Select only one)

0p: □ Enjoys walks at least twice a day (30 minutes or more each time).

1p: □ Enjoys walks and goes at least once a day (30 minutes or more each time).

2p: □ No set routine; goes for walk if taken out.

3p: □ Reluctant to walk; sometimes skips walks on bad-weather day.

4p: □ Sometimes skips walks regardless of the weather and tries to return home early.

*Approximate walking time per day and type of surface (concrete road, park, dirt dog run, beach, etc.)

Minutes per day / Location:

1. How many times a day does your dog eat? Please also briefly describe the contents of its diet. (Select only one)

0p: □ Feeds a raw diet based on knowledge of dog nutrition.

1p: □ Feeds cooked homemade food based on knowledge of dog nutrition.

2p: □ Feeds homemade food irregularly without specific planning.

3p: □ Adds homemade food to commercial dog food.

4p: □ Feeds only commercial dog food.

*Please describe the number of meals per day and the contents of the diet in detail.

Daily Times/Contents:

1. Does your dog ​​have a good appetite? Please let us know if it has any food allergies. (Select only one)

0p: □ Happily finishes two meals a day.

1p: □ Happily finishes one meal a day.

2p: □ Its appetite is inconsistent; sometimes it eats and sometimes it does not.

3p: □ Refuses to eat staple foods but eats its favorite treats.

4p: □ Refuses to eat anything at all.

*Please provide the cause of its lack of appetite, if known, and please describe the main types of food it is allergic to, if any allergies are present.

Cause/Allergy Ingredients:

1. Does the behavior of your dog when waking up or sleeping apply to any of the following? (0 or multiple answers allowed)

1p: □ The nictitating membrane sometimes takes a long time to return to its original position upon waking up.

1p: □ Less responsive to noises during sleep than before.

1p: □ Briefly opens its eyes when guests arrive, but only lifts its head before falling asleep again.

1p: □ Prefers to sleep near family members rather than in the usual sleeping place.

1p: □ Sleeps during the day but wakes up, wanders, or barks at night.

*Please describe the condition in detail, and provide the reason, if known.

Symptoms and Reason:
